# The divergence between SARS-CoV-2 and RaTG13 might be overestimated due to the extensive RNA modification

**DOI:** 10.2217/fvl-2020-0066

**Published:** 2020-03-24

**Authors:** Yue Li, Xinai Yang, Na Wang, Haiyan Wang, Bin Yin, Xiaoping Yang, Wenqing Jiang

**Affiliations:** ^1^Department of Respiratory Diseases, Qingdao Haici Hospital, PR China

**Keywords:** divergence, overestimate, RaTG13, RNA modification, SARS-CoV-2

## Abstract

**Aim:** Severe acute respiratory syndrome coronavirus 2 (SARS-CoV-2) has spread throughout the world. There is urgent need to understand the phylogeny, divergence and origin of SARS-CoV-2. **Materials & methods:** A recent study claimed that there was 17% divergence between SARS-CoV-2 and RaTG13 (a SARS-related coronaviruses) on synonymous sites by using sequence alignment. We re-analyzed the sequences of the two coronaviruses with the same methodology. **Results:** We found that 87% of the synonymous substitutions between the two coronaviruses could be potentially explained by the RNA modification system in hosts, with 65% contributed by deamination on cytidines (C-T mismatches) and 22% contributed by deamination on adenosines (A-G mismatches). **Conclusion:** Our results demonstrate that the divergence between SARS-CoV-2 and RaTG13 has been overestimated.

The spread of severe acute respiratory syndrome coronavirus 2 (SARS-CoV-2) needs to be controlled [1–3], and meanwhile its outbreak provides an opportunity for evolutionary biologists to investigate the viruses from the angle of evolution. The ultimate ambition might be finding out the origin and evolving patterns of SARS-CoV-2.

With or without much knowledge of virology, the evolutionary formula or algorithms could be easily applied to the virus sequences by using software or manual calculation. A previous study focusing on the origin and continuous evolution of SARS-CoV-2 (Tang *et al.* 2020 [4]) has an interesting finding that the synonymous substitution rate (dS) between SARS-CoV-2 and RaTG13 (one of the bat SARS-related coronaviruses) is 17%, which is 14-times the divergence between human and chimpanzee. This divergence as high as 17% is much greater than the estimation of earlier studies. The authors commented that the difference between SARS-CoV-2 and RaTG13 has been underestimated by earlier papers.

The authors’ opinion is that only the silent mutations should be used to calculate the divergence between SARS-CoV-2 and RaTG13, because these neutral sites are not affected by selection forces. By using the formula dS = 2ut, where dS represents substitution rate and u is the mutation rate, one could estimate the divergent time (t) between the two species.

Despite the terminology ‘mutation’ widely being used by evolutionary biologists, in many cases ‘mutation’ has been used in broad-sense, which represents all kinds of mismatches observed in the sequence alignment, no matter these mismatches are caused by natural mutation (such as replication errors) or other factors.

The cellular organisms have multiple RNA modification systems, which could modify any types of RNAs in the cell. Since SARS-CoV-2 and RaTG13 are coronaviruses (RNA viruses), when they infect the human cell, the RNA modification enzymes might act on the viral RNAs as they usually do to the host RNAs. Modified viral RNAs such as the methylated adenosines have been commonly observed [5–7]. Apart from the minor decorations such as methylation, two major deamination enzymes, ADAR [8] and APOBEC [9,10], are responsible for adenosine-to-inosine deamination and cytidine-to-uracil deamination, leading to an observed A-to-G and C-to-T change in the sequencing results. No matter which of SARS-CoV-2 and RaTG13 is modified, it will produce an A-G or C-T mismatch in the alignment between two viruses. In mammals, ADAR is required to fight against the infected hepatitis C virus (HCV) [11–13]. Similar to coronavirus, the HCV is a positive-strand RNA virus, and the case of ADAR acting on HCV means that the deamination on viral RNAs (thus inducing mismatches against the reference sequence) is prevalent. In other invertebrate organisms, the mismatches induced from ADAR deamination is observed in sigma virus, a negative sense RNA virus [14–16]. Evidence shows that the ADAR-modified viral RNAs are not rapidly degraded so that the ‘offspring’ of the deaminated RNA would permanently carry this mutation [11].

In the dS calculation, any observed mismatches in the sequence alignment are regarded as mutations. Of course, the software would not automatically tell the users whether a mismatch is a natural mutation caused by replication error or an RNA modification site.

However, for DNA organisms like humans, the classic definition of mutation rate should mainly (perhaps not absolutely) refer to the replication error rate of DNA. For SARS-CoV-2, the mutation rate should mainly refer to the RNA replication error rate. Accordingly, the calculation of dS should only include the natural mutations introduced during RNA replication rather than the RNA-to-RNA mismatch sites caused by RNA modification system. The replication error rate should be very low while the occurrence of RNA modification could appear in any virus RNA which is exposed to the host's deamination enzymes. The RNA modification rate could be higher than RNA replication rate for orders of magnitude. The phenomenon that the viral RNAs or even proteins are modified by host cells is not rare at all [13,17] so that this issue should be considered when studying the divergence of RNA viruses.

Our idea is that when checking the sequence alignment between SARS-CoV-2 and RaTG13, if one found that plenty of the synonymous substitutions could be potentially explained by C-to-T deamination or A-to-G deamination then the actual divergence between SARS-CoV-2 and RaTG13 might have been overestimated by many times. We re-emphasize that we only say the C-T and A-G mismatches could be potentially explained by RNA modification but not definitely caused by RNA modification. The aim is to rationally estimate the real divergence between the two RNA viruses.

## Materials & methods

We downloaded the sequences of SARS-CoV-2 and RaTG13 from GeneBank and aligned the coding sequences with MUSCLE [13]. The 11 nonredundant ORFs are annotated with names (such as *M*, *N*, *ORF1AB*) so that we put the two ortholog genes into a file and run the sequence alignment. The length of each ORF (number of amino acids) and the aligned length of each ORF are given in Table 1. For example, we put the two sequences of SARS-CoV-2 ORF10 and RaTG13 ORF10 into one file and run MUSCLE with default parameter. Then the output file would give us the aligned sequences of these two ORFs. From Table 1, we could see that the ORFs in two virus species are almost of the same length so that the parameters hardly affect the alignment results. We manually extract each codon in the alignment file using our own python script. The unaligned regions are gaps. As shown in Table 1, only ORF1AB have two triplets (codons) unaligned, and the other regions and other ORFs are well aligned. Next, most of the aligned regions are identical. The nonidentical regions are either missense or synonymous mutations. To help readers understand the process of extracting mutations (mismatches) from the alignment, we listed the first ten missense and synonymous mutations of ORF1AB in Tables 2 & 3, respectively. From Tables 2 & 3, we already see prevalent C-T mismatches.

**Table 1.** The length and aligned length of each ORF of SARS-CoV-2.

ORF ID	Length (amino acids)	Aligned length in RaTG13
E	75	75
M	222	222
N	419	419
ORF10	38	38
ORF1AB	7095	7093
ORF3A	275	275
ORF6	61	61
ORF7A	121	121
ORF7B	43	43
ORF8	121	121
S	1273	1273

**Table 2.** The first ten missense mutations in ORF1AB.

Position	SARS-CoV-2	RaTG13	Amino acid (SARS-CoV-2)	Amino acid (RaTG13)	Mismatch (nondirectional)
38	GTC	GCT	Val	Ala	C-T
110	CAT	TAT	His	Tyr	C-T
114	ATA	ACA	Ile	Thr	C-T
117	GCT	GTT	Ala	Val	C-T
172	GAA	GAT	Glu	Asp	A-T
280	ATA	ACA	Ile	Thr	C-T
376	TCA	CCA	Ser	Pro	C-T
395	ACC	CCC	Thr	Pro	A-C
417	CAT	TAC	His	Tyr	C-T
424	GTT	ATT	Val	Ile	A-G

**Table 3.** The first ten synonymous mutations in ORF1AB.

Position	SARS-CoV-2	RaTG13	Amino acid (SARS-CoV-2)	Amino acid (RaTG13)	Mismatch (nondirectional)
20	GTT	GTC	Val	Val	C-T
59	GGC	GGT	Gly	Gly	C-T
74	TCG	TCT	Ser	Ser	G-T
82	GGT	GGC	Gly	Gly	C-T
92	CTC	CTT	Leu	Leu	C-T
97	TAC	TAT	Tyr	Tyr	C-T
104	CTT	CTC	Leu	Leu	C-T
138	GCC	GCT	Ala	Ala	C-T
142	TCA	TCG	Ser	Ser	A-G
169	GTT	GTC	Val	Val	C-T

It is possible that sometimes the mutation may be lethal, producing shortened protein if TAA is produced instead of CAA. We scanned the 11 nonredundant ORFs in SARS-CoV-2 and RaTG13. We did not find any internal stop codons in these ORFs.

For the multiple alignment incorporating other virus species ZXC21, ZC45 and BM48-31, we aligned the ORFs with the same method. Together with SARS-CoV-2 and RaTG13, we put the orthologous ORF of the five species into one file and run MUSCLE. The output alignment file was manually inspected. Each codon located in the ORFs were simply extracted by our own python scripts. The results of aligning SARS-CoV-2 and RaTG13 and the results calculated from aligning five species were compared. The relative alignment and mismatch profiles between SARS-CoV-2 and RaTG13 were found to be identical under two sets of strategies.

The ID of SARS-CoV-2 is NC_045512. The link of SARS-CoV-2 ORF1AB (coding sequence) is: https://www.ncbi.nlm.nih.gov/nuccore/NC_045512.2?from=266&to=21555&report=fasta

The ID of RaTG13 is MN996532. The link of RaTG13 genome is: https://www.ncbi.nlm.nih.gov/nuccore/MN996532.1/?report=fasta

The beginning of SARS-CoV-2 ORF1AB is ‘ATG|GAG|AGC|CTT|GTC’, the end of SARS-CoV-2 ORF1AB is ‘GAT|GTT|CTT|GTT|AAC|AAC|TAA’. By manually searching ‘ATGGAGAGCCTTGTC’ and ‘GATGTTCTTGTTAACAACTAA’ in the RaTG13 genome sequence, we can anchor and extract the ORF1AB in the RaTG13 genome. The ORF1AB CDS alignment between SARS-CoV-2 and RaTG13 is provided in Supplementary Table 1. As we can see, most codons are identical. The nonidentical codons mostly have synonymous mutations.

## Results

### Substitutions between SARS-CoV-2 & RaTG13

We aligned the ORFs of SARS-CoV-2 and RaTG13 and manually extracted the codons in the alignment file (see Materials and methods). The statistics of the alignment results (Table 1) show that most of the ORFs are well aligned and only ORF1AB has two gaps. From the 9.7 thousand codons in the ORFs, we totally obtained 1076 nonidentical codon positions between SARS-CoV-2 and RaTG13, 931 of which encode the same amino acid (synonymous) and 145 of which encode different amino acids (missense). That is to say, there are 931 synonymous substitutions and 145 missense substitutions between SARS-CoV-2 and RaTG13. The other ORF regions (90%) are identical between SARS-CoV-2 and RaTG13.

Among the 9.7 thousand codons in the 11 nonredundant SARS-CoV-2 ORFs, the content of C and T is 51.2%. However, among the 931 codons with synonymous substitutions, the content of C and T is 56.1%, and the difference is significant using Chi-square test (p = 5.7E-3). It proves that the occurrence of synonymous substitutions is nonrandom and it tends to take place on codons containing C or T.

### 87% of the synonymous substitutions are C-T or A-G mismatches

**Figure 1.**
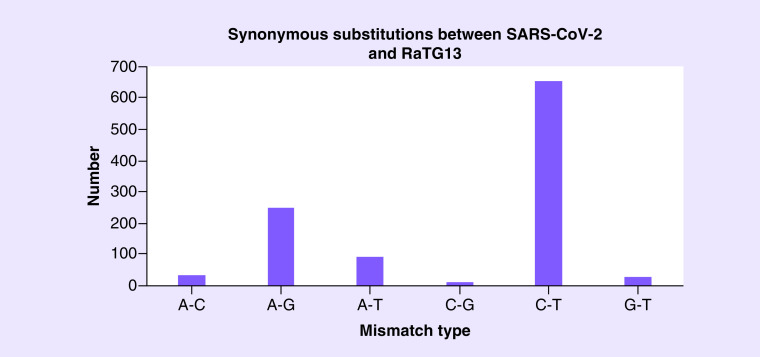
The numbers of mismatch types on synonymous substitution sites between SARS-CoV-2 and RaTG13.

We checked the 1076 substitution sites between SARS-CoV-2 and RaTG13, 84.4% of the mutations are A-G or C-T mismatches (61.0% C-T mismatches and 23.4% A-G mismatches). Among the 931 synonymous substitution sites (Figure 1), 86.7% of them are A-G or C-T mismatches (64.9% C-T mismatches and 21.8% A-G mismatches). This mismatch spectrum resembles the enrichment of C-to-T(U) deamination and A-to-G(I) deamination. Nearly 87% of the observed synonymous ‘mutations’ between SARS-CoV-2 and RaTG13 could be potentially explained by the RNA modification systems in host cells.

To help readers understand how the mismatches were extracted from the alignment file, we listed the first ten missense and synonymous mutations in ORF1AB, respectively (Tables 2 & 3). We have said that 90% of the aligned regions is identical and the nonidentical codons usually differ with a single nucleotide. In Table 2, seven out of the ten missense substitutions were C-T mismatches. In Table 3, eight out of the ten synonymous substitutions were C-T mismatches. Given the high similarity of the SARS-CoV-2 and RaTG13 sequences, these mismatches may not be caused by mis-alignment.

One may also be concerned whether the alignment and mismatch profile is different when using multiple virus species to run the alignment. We downloaded the ORFs of other SARS-related coronaviruses ZXC21, ZC45, and BM48-31 (see Materials and methods). We found that using multiple species does not affect the aligned regions between SARS-CoV-2 and RaTG13. Although additional gaps are introduced in the alignment, the relative position between SARS-CoV-2 and RaTG13 remains the same. So, the mismatches between SARS-CoV-2 and RaTG13 are not affected by different alignment strategies. The prevalence of C-T and A-G mismatches is robust.

### Mismatch profile excluding the protease digestion sites in ORF1AB

The ORF1AB (pp1AB) would be cleaved into multiple proteins (nsp1-16) by protease. The cleavage sites are LQS and LQA sequences [18,19]. We could not simply call ORF1AB as one gene, so it is rational to exclude the mutations in digestion sites in the divergence analyses. We checked the mutations in the LQS and LQA regions. We only found one case. Amino acids 4252-4254 is Leu-Gln-Ala, and the Leu codon is CTA in SARS-CoV-2 and TTA in RaTG13. This single C-T mismatch in digestion regions does not affect the overall mismatch profile. This also proves that the amino acid sequences of digestion sites might be highly conserved to avoid the loss of protease recognition. Again, our finding of prevalent C-T and A-G mismatches is robust.

### ORF1AB & S contribute most of the mismatches

It is necessary to provide the influence of the tested number of genes on the estimated divergence. As seen in Table 1, ORF1AB and S are the longest ORFs. They contribute most of the mismatches if we look at the mismatch profile in all the ORFs. Here we list the dS values calculated by Tang *et al.* [4] and the percent of mismatches potentially explained by RNA modification (Table 4). ORF1AB, S, and the other ORFs are listed separately. Clearly, the choice of tested genes does not severely affect the pattern. In all genes, 87% mutations could be (potentially) explained by modified RNA. In ORF1AB, 89% mutations could be (potentially) explained by RNA modification. In S, 78% mutations could be (potentially) explained by modified RNA. In the remaining ORFs, 91% mutations could be (potentially) explained by modified RNA. Presume that 91% of the mismatches are caused by RNA modification, then the dS value is overestimated for more than tenfolds. The S ORF has a pretty high dS value, so it is especially necessary to question if the modification system contributes to the divergence.

**Table 4.** dS values and the fold of overestimation.

ORF	dS (Tang *et al.*)	C-T mismatch	A-G mismatch	Explained by modification (upper bound)	Fold of overestimation of dS (upper bound)
All	0.17	65%	22%	87%	7.7
ORF1AB	0.152	67%	22%	89%	9.2
S	0.321	59%	19%	78%	4.5
Other	Not provided	64%	27%	91%	10.9

## Discussion

One argument is that in the alignment between SARS-CoV-2 and RaTG13 we did not use an outgroup species so that the direction of the mutation is uncertain. Yes, that is true. We do not worry about the ancestral state. SARS-CoV-2 and RaTG13 are RNA viruses. As long as we observe a C-T or A-G mismatch in the sequence alignment between them, we could speculate that the C-to-T or A-to-G deamination might have occurred in one of the two virus species.

Note that we only say 87% of the mutations could be potentially explained by RNA modification, rather than 87% of them are definitely caused by RNA modification. From the sequence alignment alone, it is impossible to know whether the mismatch is a ‘*de novo*’ mutation or an RNA modification site. The software would not tell users what has caused this mismatch since it is technically indistinguishable. Improving the parameters only makes alignment more accurate but does not tell us the origin of the mismatch.

As understood by common researchers, the definition of dS between RNA viruses mainly (but not absolutely) refers to the natural mutations introduced by RNA replication error rather than the RNA modification sites caused by host cells. The RNA modification rate is many times higher than the replication error rate. This fact is consistent with our notion that the divergence between RNA viruses is overestimated.

According to our results, potentially 87% of the synonymous substitutions between SARS-CoV-2 and RaTG13 could be caused by RNA modification system in hosts. The remaining 13% of the substitutions should be genuine interspecific mutations as they could not be explained by known RNA modification types. The claimed dS = 0.17 should have been overestimated. The upper bound of overestimation is 1/0.13 = 7.7-times so that the lower bound of the dS value is 0.17/7.7 = 0.022.

Indeed, if the authors argue that the definition of dS itself already included any mutation types such as those RNA modification sites then the dS value of 17% would be valid. However, this definition of dS is not what we commonly understand, and the authors should have pointed this out in their article. Again, adjusting the parameters of any software only makes the alignment more accurate but is not helpful in determining whether the observed mismatches are modified RNA or the natural mutation introduced during RNA replication. A rational way to avoid a wrong and misleading conclusion is to calculate the upper bound and lower bound of the divergence value. Anyway, the currently proposed divergence (dS = 17%) between SARS-CoV-2 and RaTG13 has been severely overestimated. We appeal that when calculating dN and dS between RNA viruses, the RNA modification should be taken into account.

The limitation of our study is that we were currently unable to provide experimental evidence for the modification on viral RNAs although this phenomenon is not new for virologists. At the same time, neither did Tang *et al.* [4] provide evidence to prove that the mismatches in the alignment are not caused by RNA modification. Since both sides lack experimental evidence, it is reasonable to think about this dilemma from the angle of maximum likelihood. That is, if the mismatch sites between SARS-CoV-2 and RaTG13 are really introduced by accumulation of RNA replication errors, they should not exhibit an excessive number of C-T and A-G mismatches (in that case the mutation types should be random).

Another limitation of our work is that we did not give an estimation of the real divergence value. As we have stated, the RNA modifications and normal mutation sites are technically indistinguishable. We only say that the proposed 17% divergence is higher than the real value but we still do not know what the real value is. Promisingly, experts in mutations could estimate the relative abundance of each type of mismatches and give a reasonable value of the divergence between SARS-CoV-2 and RaTG13.

## Conclusion

Since we found 87% of the synonymous substitution sites between SARS-CoV-2 and RaTG13 could be potentially explained by RNA modification system in host cells, we are strongly concerned that the previously defined divergence between SARS-CoV-2 and RaTG13 has been overestimated.

**Summary points**

- The outbreak of severe acute respiratory syndrome coronavirus 2 (SARS-CoV-2) has caused severe damage to the world.
- It is necessary to understand the origin and evolution patterns of SARS-CoV-2.
- A previous study claimed that SARS-CoV-2 and RaTG13 have 17% divergence on synonymous sites.
- We aligned the coding sequences of SARS-CoV-2 and RaTG13, and checked the substitution sites between them.
- The substitution sites are CT-enriched compared with background.
- Potentially 87% of the synonymous substitutions between SARS-CoV-2 and RaTG13 could be explained by RNA modification system in hosts.
- The divergence between SARS-CoV-2 and RaTG13 has been overestimated.
- The calculation of dN or dS between RNA viruses should take the RNA modification into consideration.
